# RETRACTION: The Impact of Different Antibiotic Injection Regimens on Patients With Severe Infections: A Meta‐Analysis

**DOI:** 10.1111/iwj.70592

**Published:** 2025-04-21

## Abstract

**RETRACTION:** LiD. , MoK. , LiangB. , HuangY. , TanX. , WangZ. , and YangX. , “The Impact of Different Antibiotic Injection Regimens on Patients With Severe Infections: A Meta‐Analysis,” International Wound Journal 21, no. 1 (2024): e14514, 10.1111/iwj.14514.38272804 PMC10791546

The above article, published online on 16 January 2024, in Wiley Online Library (http://onlinelibrary.wiley.com/), has been retracted by agreement between the journal Editor in Chief, Professor Keith Harding; and John Wiley & Sons Ltd. Following an investigation by the publisher, all parties have concluded that this article was accepted solely on the basis of a compromised peer review process. The editors have therefore decided to retract the article. The authors did not respond to our notice regarding the retraction.

**RETRACTION:** D. Li , K. Mo , B. Liang , Y. Huang , X. Tan , Z. Wang , and X. Yang , “The Impact of Different Antibiotic Injection Regimens on Patients With Severe Infections: A Meta‐Analysis,” International Wound Journal 21, no. 1 (2024): e14514, 10.1111/iwj.14514.38272804 PMC10791546

The above article, published online on 16 January 2024, in Wiley Online Library (http://onlinelibrary.wiley.com/), has been retracted by agreement between the journal Editor in Chief, Professor Keith Harding; and John Wiley & Sons Ltd. Following an investigation by the publisher, all parties have concluded that this article was accepted solely on the basis of a compromised peer review process. The editors have therefore decided to retract the article. The authors did not respond to our notice regarding the retraction.

